# Cytoskeleton structure and total methylation of mouse cardiac and lung tissue during space flight

**DOI:** 10.1371/journal.pone.0192643

**Published:** 2018-05-16

**Authors:** Irina V. Ogneva, Sergey S. Loktev, Vladimir N. Sychev

**Affiliations:** 1 Cell Biophysics Lab, State Scientific Center of the Russian Federation Institute of Biomedical Problems of the Russian Academy of Sciences, Moscow, Russia; 2 I. M. Sechenov First Moscow State Medical University, Moscow, Russia; University of California Berkeley, UNITED STATES

## Abstract

The purpose of this work was to evaluate the protein and mRNA expression levels of multiple cytoskeletal proteins in the cardiac and lung tissue of mice that were euthanized onboard the United States Orbital Segment of the International Space Station 37 days after the start of the SpaceX-4 mission (September 2014, USA). The results showed no changes in the cytoskeletal protein content in the cardiac and lung tissue of the mice, but there were significant changes in the mRNA expression levels of the associated genes, which may be due to an increase in total genome methylation. The mRNA expression levels of DNA methylases, the cytosine demethylases Tet1 and Tet3, histone acetylase and histone deacetylase did not change, and the mRNA expression level of cytosine demethylase Tet2 was significantly decreased.

## Introduction

Under microgravity conditions, there are different negative changes in the skeletal, muscle and cardiovascular systems. Animal experiments have facilitated the investigation of the structural changes in the myocardium and skeletal muscles that arise during space flights.

Previous research has revealed atrophic changes in the skeletal muscles of rats after space flight [1–4]. Moreover, early readaptation leads to more pronounced negative changes in the structure of muscle fibers than those observed immediately after the flight [5–7].

A fluid shift in the cranial direction [8, 9] and changes in systolic output [10–12] that are observed under conditions of weightlessness, could lead to functional changes in the cardiovascular system. Nevertheless, despite the decrease in the functional capabilities of the myocardium under conditions of microgravity, structural changes in the myocardium have not been observed after landing [1]. Data obtained from murine cardiomyocytes after the flight of the BION-M Biosatellite No. 1 (dissection was carried out within 13–16.5 hours after landing) indicated that the levels of cytoskeletal proteins (beta- and gamma-actin, actin-binding proteins, desmin, and tubulin), as well as the expression levels of the genes encoding those proteins, were significantly changed after a 30-day space flight [13]. In the same experiment, Povysheva T.V. et al. showed that there were also changes in the structure of the myelinated fibers [14].

Similar effects were also observed in cells cultivated under conditions simulating the effects of microgravity. It was shown under such conditions, a change in the structure of microfilaments and microtubules occurs in mesenchymal stem and embryonic stem cells, leading to a change in the cellular shape and differentiation potential [15–21]. Moreover, it was shown by live-cell imaging that poorly differentiated follicular thyroid cancer cells (FTC-133) developed less aggressive phenotypes under microgravity conditions [22]. However, recently obtained data indicate that under conditions of real weightlessness, the rearrangements of F-actin and intermediate filaments were not observed in macrophages [23].

The effects of microgravity on gene expression have been documented in mammalian cells [24, 25] and other model organisms, such as yeast and bacteria [26–28]. However, the mechanisms underlying these effects are still unclear.

DNA methylation is one of the main epigenetic factors [29]. Although methylation patterns are established during development, environmental conditions can also induce changes in these patterns [30]. Thus, Weaver I.C. et al. showed that different maternal care leads to different methylation patterns of the promoter region of the glucocorticoid receptor gene (and correlates with its expression) in young rats and that these effects persist even into adulthood [31].

To date, very few studies have been dedicated to the analysis of DNA methylation levels in space flight conditions or the modeling of the subsequent effects on Earth.

Ou X. et al. [32] conducted experiments with rice plants during space flight and only showed hypermethylation that correlated with the transcription levels of genes. In contrast, Singh K.P. et al. [33] studied human T-lymphocyte cells, which were grown in a rotary cell culture system, and showed hypomethylation of the DNA. Chowdhury B. et al. [34] used TK6 human lymphoblastoid cells and ground-based simulation of microgravity with an HARV Rotary Cell Culture System for 48 hours and revealed that simulated microgravity induced alterations in the methylome (~60% of the differentially methylated regions or DMRs were hypomethylated and ~92% of the differentially hydroxymethylated regions or DHMRs were hyperhydroxymethylated).

Therefore, there is a substantial amount of conflicting data regarding the effects of space flight on cell structure and the regulation of transcription, which may be due to varying periods between landing and biomaterial fixation.

We suggest that for cardiac tissue, the early period of readaptation is accompanied by a decrease in volume load due to fluid shift. It could lead to the changes of the cortical cytoskeleton structure that were observed after the BION-M1 mission [13]. Therefore, we were interested in comparing the data from that biomaterial with data from biomaterial fixed under weightlessness conditions to avoid landing and readaptation effects. It can be assumed that such a strategy could provide us with a unique possibility to find methods for future investigations with the aim of protecting humans subjected to long-term space flight and the subsequent readaptation to Earth’s gravity and possibly that of Mars. The Rodent Research-1 experiment provided such an opportunity.

In this paper, we present the results of a study of mouse heart and lung tissues obtained from the Rodent Research-1 experiment, where the biomaterial was fixed under space flight conditions in the US Orbital Segment of the ISS. We tested the protein and mRNA levels of the same cytoskeletal proteins (beta-actin, gamma-actin, alpha-actin-1, alpha-actinin-4, tubulin, and desmin) and compared the results to the previously observed data. In addition, after observing the relative changes in mRNA expression, we analyzed one of the possible factors affecting gene expression, total DNA methylation and its regulation.

## Materials and methods

### Experimental design

C57Bl6/J mice were used (Jackson Laboratory, Bar Harbor, ME) in the Rodent Research-1 (RR-1) experiment. The date of birth of the mice was June 3, 2014. The adaptation period was approximately 4 weeks. The age of the mice at the time of the launch was 16 weeks. The animals were placed in a transport container (10 mice in each container divided into 2 parts) on September 19, 2014. For two days, the transport container remained in the Dragon spacecraft capsule at the launch table. The SpaceX-4 spacecraft was launched on September 21, 2014. The animals were transferred from the transport container to the animal habitat block on the ISS (5 mice per block divided into 2 parts) on September 25, 2014.

The biomaterial sampling schedule was as follows.

22 September 2014: Animals of the basal control group (B) were euthanized at the laboratory shortly after the launch.

28 October 2014: Animals of the flight group (F) were euthanized onboard the ISS 37 days after the launch (after a 33-day stay on the ISS with food and water provided *ad libitum*).

31 October 2014: Animals of the vivarium control group (V), which were housed in standard vivarium conditions, were euthanized.

01 November 2014: Animals of the ground control group (G) were euthanized at the laboratory after spending the same amount of time as the flight duration in the chamber simulating the conditions of the environment on the ISS located in the John F. Kennedy Space Center (USA).

The mice used in this study were immediately frozen after euthanasia without biomaterial isolation. The time from cervical dislocation to the freezing of the carcasses was approximately 2 minutes. The carcasses were wrapped in two layers of foil and placed into plastic bags, which were then inserted into bags intended for storage at low temperatures (packed with 3 pre-chilled ice packs). The bags were stored in the MELFI freezer (-80°C) located in the USOS of the ISS until the return to the Earth. The biomaterial was delivered to Earth by SpaceX-5 (landed 11 February 2015). The schedule of the work with the animals in the ground and vivarium control groups matched the schedule of the work with the animals on the flight.

All animal procedures were approved by the Ames Institution Animal Care and Use Committee (protocol CAS-13-001-Y1, approved May 13, 2014, Ames, USA).

The organs were isolated from the frozen carcasses on Earth by our colleagues from the Ames Research Center of NASA. The dissections were performed after defrosting the carcasses to +4°C. The dissection procedure used for the control groups was identical to that used for the flight group. After isolation, the right side of the heart and one lung were frozen in liquid nitrogen and the left ventricle of the heart and the second lung were placed in RNAlater Stabilization Solution (Qiagen, Germany). We investigated the biomaterial of 5 animals from each group (n = 5 for each group). All tests were carried out at least three times for each sample.

The obtained samples, according to the NASA-Roscosmos protocol “Utilization Sharing Plan On-Board ISS” (signed on July 18, 2013), were delivered to the Russian Federation in dry ice without defrosting, which was controlled by temperature detectors.

### Determination of the protein content levels using gel electrophoresis followed by immunoblotting

One of the lungs and the right side of the heart were frozen in liquid nitrogen for subsequent determination of the content of several cytoskeletal proteins. Tissue extracts were used to prepare the cytoplasmic and membrane fractions of the proteins [35]. Denaturing electrophoresis on polyacrylamide gels was performed using the Laemmli method and a Bio-Rad system (USA). Based on the measured concentration, an equal amount of protein was placed into each well, separated by electrophoresis, and transferred to a nitrocellulose membrane [36]. The following specific primary monoclonal antibodies were used at the dilutions recommended by the manufacturers to determine the levels of each protein: mouse antibodies against beta-actin (diluted 1:300), gamma-actin (diluted 1:100), alpha-actinin-1 (diluted 1:100), alpha-actinin-4 (diluted1:100), desmin (diluted 1:300) (Santa Cruz Biotechnology, Inc., USA), and rabbit antibodies against beta-tubulin (diluted 1:1000) (Pierce Thermo Scientific, Inc., USA). Biotinylated goat antibodies were used as the secondary antibodies to detect rabbit IgG (Jackson ImmunoResearch Lab, Inc., USA) at a dilution of 1:10,000; to detect mouse IgG (Sigma, Germany), the secondary antibodies were used at a dilution of 1:20,000. Afterwards, all the membranes were treated with a streptavidin solution conjugated to horseradish peroxidase (Sigma, Germany) that was diluted 1:10,000. Protein bands were detected using 3,3’-diaminobenzidine (Merck, USA). The ImageJ program was used to analyze the obtained data.

### Determination of mRNA content using quantitative PCR method

Total RNA was isolated from the left ventricle of the heart and the lungs, stabilized in RNAlater Stabilization Solution (Qiagen, Germany), and isolated using the RNeasy Micro Kit (Qiagen, Germany) according to the manufacturer’s instructions to perform the target analysis of the mRNA content of the genes that encode a number of cytoskeletal and metabolic proteins, as well as different cytosine methylases and demethylases, and histone acetylase and deacetylase. Reverse transcription was performed using d(T)_15_, and 500 ng of RNA was used as an inoculum, controlled by measuring the concentration. After reverse transcription, the amount of cDNA was measured. To avoid artifacts, all samples had at least three replicates. Real-time PCR was performed using the primers selected via the Primer3Plus program (Table 1) to assess the expression levels of the studied genes. The 2(-Delta Delta C(T)) method [37] was used to determine the fold change.

**Table 1:** Primer sequences and product sizes.

Gene	Primer sequence, forward/reverse (5'…3')	Product size, bp
*Actb* (beta-actin)	*gctgcgttttacaccctttc/ gtttgctccaaccaactgct*	218
*Actg* (gamma-actin)	*ctggtggatctctgtgagca/ tcaggagggaagaaaccaga*	184
*Arpc3* (actin-related protein 2/3 complex, subunit 3)	*acaggaggatgaaacgatgc/ tcacaaagcaagtccaccac*	125
*Svil* (supervillin)	*tcggaaaccaagacgctatc/ accactggcaattctgaacc*	132
*Lcp1* (L-plastin)	*gaagggatcgtcaaacttgc/ tatggttctgaggggattgg*	130
*Actn1* (alpha-actinin 1)	*ggtcagcagcaacctcctc/ tctttctccaccttctctcca*	167
*Actn4* (alpha-actinin 4)	*accctgaacagactcccttg/ gatcgacaagcctccatctc*	168
*Des* (desmin)	*gtgaagatggccttggatgt/ cgggtctcaatggtcttgat*	182
*Tubb2b* (beta-tubulin 2B)	*ggcagcaagaagctaacgag/ cgaacacgaagttgtctggc*	302
*Cct5* (chaperonin containing Tcp-1, subunit 5—epsilon)	*caaacgggctggataagatg/ tcctgggatttggacagttc*	136
*Cct7* (chaperonin containing Tcp-1, subunit 7—eta)	*tgtgaccgtgaagaagcaag/ gcatcatcacagcatcaacc*	137
*Cycs* (cytochrome *c*)	*ccaaatctccacggtctgtt/ tctgccctttctcccttctt*	190
*Gapdh* (glyceraldehyde 3-phosphate dehydrogenase)	*acccagaagactgtggatgg/ acacattgggggtaggaaca*	172
*Dnmt1* (S-phase methylation)	*ccggaaactcacttggacga/ tttggcagctggatctctgg*	90
*Dnmt3A* (*de novo* methylation)	*agagcgctttgactccacat/ ggaccaggaaaaacaaacga*	150
*Tet1* (cytosine demethylase)	*gtgtgggtcgatggctctat/ cttattcccaccaccgctaa*	208
*Tet2* (cytosine demethylase)	*gttctcaacgagcaggaagg/ tgagatgcggtactctgcac*	185
*Tet3* (cytosine demethylase)	*ttctatccgggaactcatgg/ ccaggccaggatcaagataa*	226
*Hat1* (histone aminotransferase 1)	*agagtgccgtggagaagaaa/ tttcatcatccccaaagagc*	150
*Hdac1* (histone deacetylase 1,2,3,4,6,9)	*ccatgaagcctcaccgaat/ caaacaccggacagtcctca*	226

### Determination of DNA total methylation level using restriction analysis (MspI/HpaII)

To determine the level of methylation, total DNA was isolated from the tissue of the left ventricle of the heart and lungs that had been placed in RNAlater Stabilization Solution (Qiagen, Germany) using a DNA extraction kit (Syntol, Russian Federation) based on the phenol/chloroform method according to the manufacturer's instructions. For the total CpG methylation analysis at the 5’-CCGG-3’ locus, an EpiJET Methylation Analysis Kit (MspI/HpaII) (Thermo Scientific, USA) was used according to the manufacturer’s instructions. When the internal CpG in the 5’-CCGG-3’ tetranucleotide is methylated, cleavage by Epi HpaII is blocked, but cleavage by Epi MspI is not affected. During the restriction digest, 1 μg of genomic DNA and 2 μg of unmethylated plasmid DNA pUC DNA/SmaI and fully methylated plasmid DNA mpUC DNA/SmaI were used in the absence of DNA degradation in the buffer as a control for the restriction and efficiency of the MspI and HpaII enzymes. The restriction results analysis was carried out in a 1% agarose gel with a size marker, FastRuler Middle Range DNA Ladder (Thermo Scientific, USA), and the results were processed with the Image Lab program (Bio-Rad, USA), with normalization of the total content of the undigested genomic DNA in the corresponding sample.

### Statistical analysis

The results obtained from the determination of the protein and mRNA expression levels and the methylation level were analyzed using ANOVA and post hoc t-test, and a significance level of p < 0.05 was applied to assess the significance of differences between the groups. The data are presented as the M±SE, where M represents the mean, and SE represents the standard error of the mean. All data used to build graphs were uploaded to the Dryad Digital Repository (doi:10.5061/dryad.h2j2466).

## Results

### Cytoskeletal protein content in the membrane and cytoplasmic fractions

**Fig 1:**
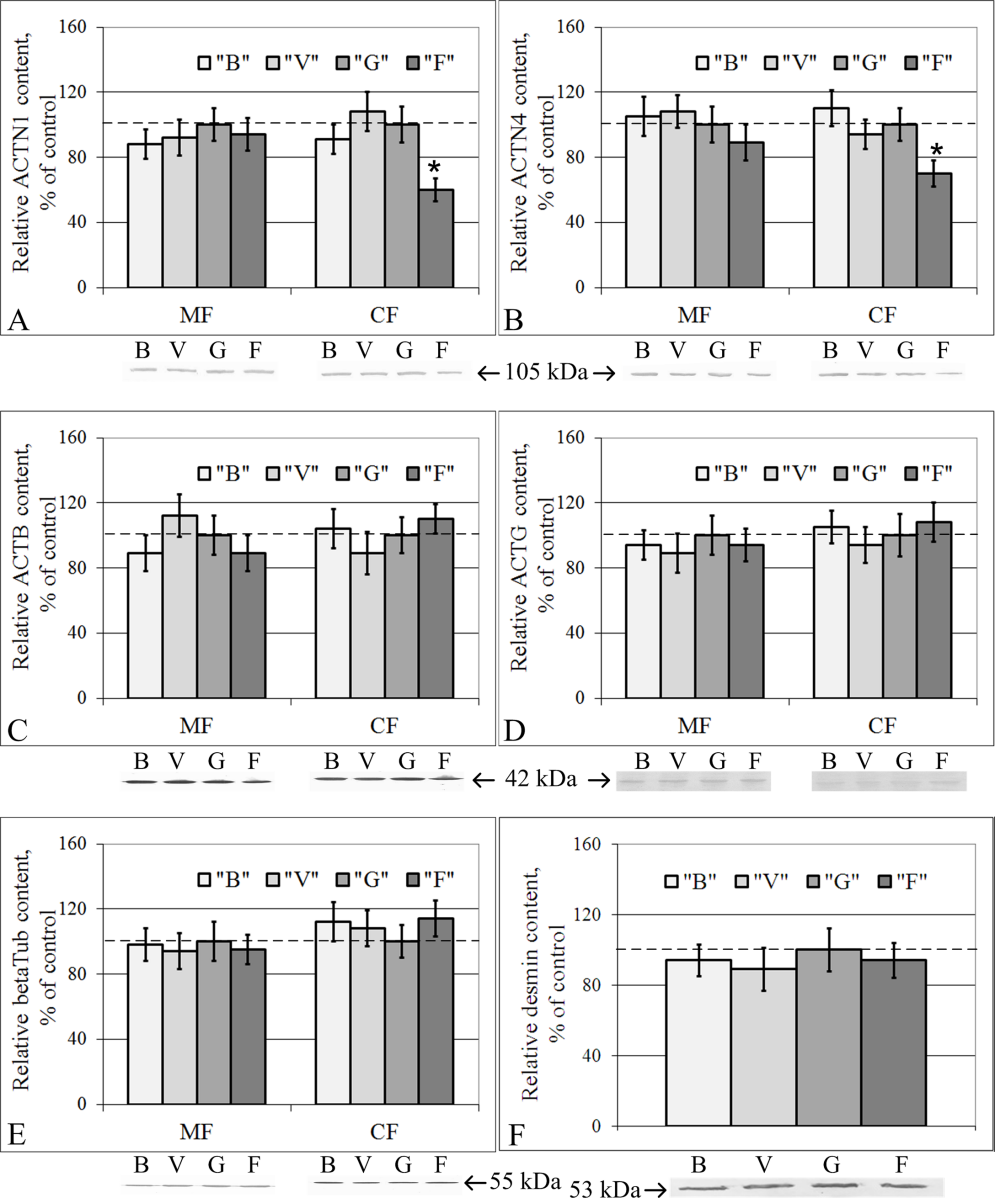
Relative contents of cytoskeletal proteins in the membrane (MF) and cytoplasmic (CF) fractions of cardiomyocytes with typical Western blot images. (A) Alpha-actinin-1. (B) Alpha-actinin-4. (C) Beta-actin. (D) Gamma-actin. (E) Beta-tubulin. (F) Desmin. “B”–basal control group, “V”–vivarium control group, “G”–ground control group (level marked by dotted line), “F”–flight group. *–p < 0.05 in comparison with group “G”. The values used to build graphs represented in the S1 Table. There were no changes in the cytoskeletal proteins contents in the membrane fraction of the cardiac tissue. In the cytoplasmic fraction, alpha-actinin-1 and alpha-actinin-4 protein content decreased during space flight.

The relative contents of beta-actin, gamma-actin, alpha-actinin-1, alpha-actinin-4, and beta-tubulin in the membrane fraction of the right ventricle of the heart were similar among the B, V, G and F groups (Fig 1). In the cytoplasmic fraction, the relative protein contents of beta-actin (Fig 1C), gamma-actin (Fig 1D), beta-tubulin (Fig 1E), and desmin (Fig 1F) were also unchanged in groups B, V, G and F. At the same time, the relative protein contents of alpha-actinin-1 (Fig 1A) and alpha-actinin-4 (Fig 1B) decreased by 40% (p < 0.05) and 30% (p < 0.05), respectively, in group F compared to group G.

**Fig 2:**
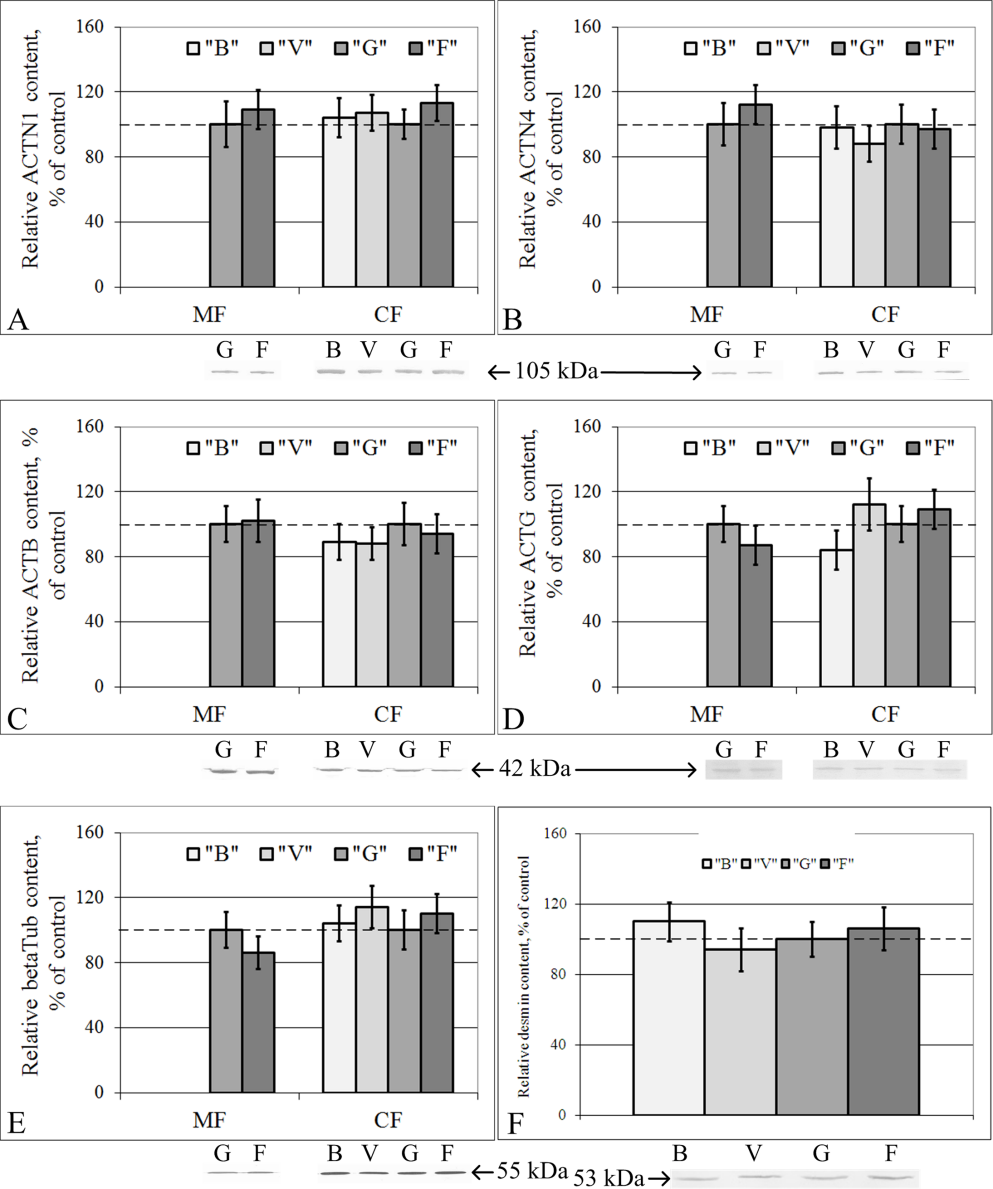
Relative contents of cytoskeletal proteins in the membrane (MF) and cytoplasmic (CF) fractions of lung cells with typical Western blot images. (A) Alpha-actinin-1. (B) Alpha-actinin-4. (C) Beta-actin. (D) Gamma-actin. (E) Beta-tubulin. (F) Desmin. “B”–basal control group, “V”–vivarium control group, “G”–ground control group (level marked by dotted line), “F”–flight group. The values used to build graphs represented in the S2 Table. There were no changes of cytoskeletal proteins contents in the membrane and cytoplasmic fractions of the lung tissue.

The relative protein contents of different cytoskeletal components (components of microfilaments, microtubules and intermediate filaments) in the cytoplasmic fraction of the lung tissue was similar in all the study groups (B, V, G and F) (Fig 2). We failed to isolate total protein of the membrane fraction of lung tissue that was of adequate quality in groups B and V, but in this case, no differences between groups G and F were found (Fig 2).

### The mRNA contents of genes that encode some cytoskeletal and metabolic proteins

**Fig 3:**
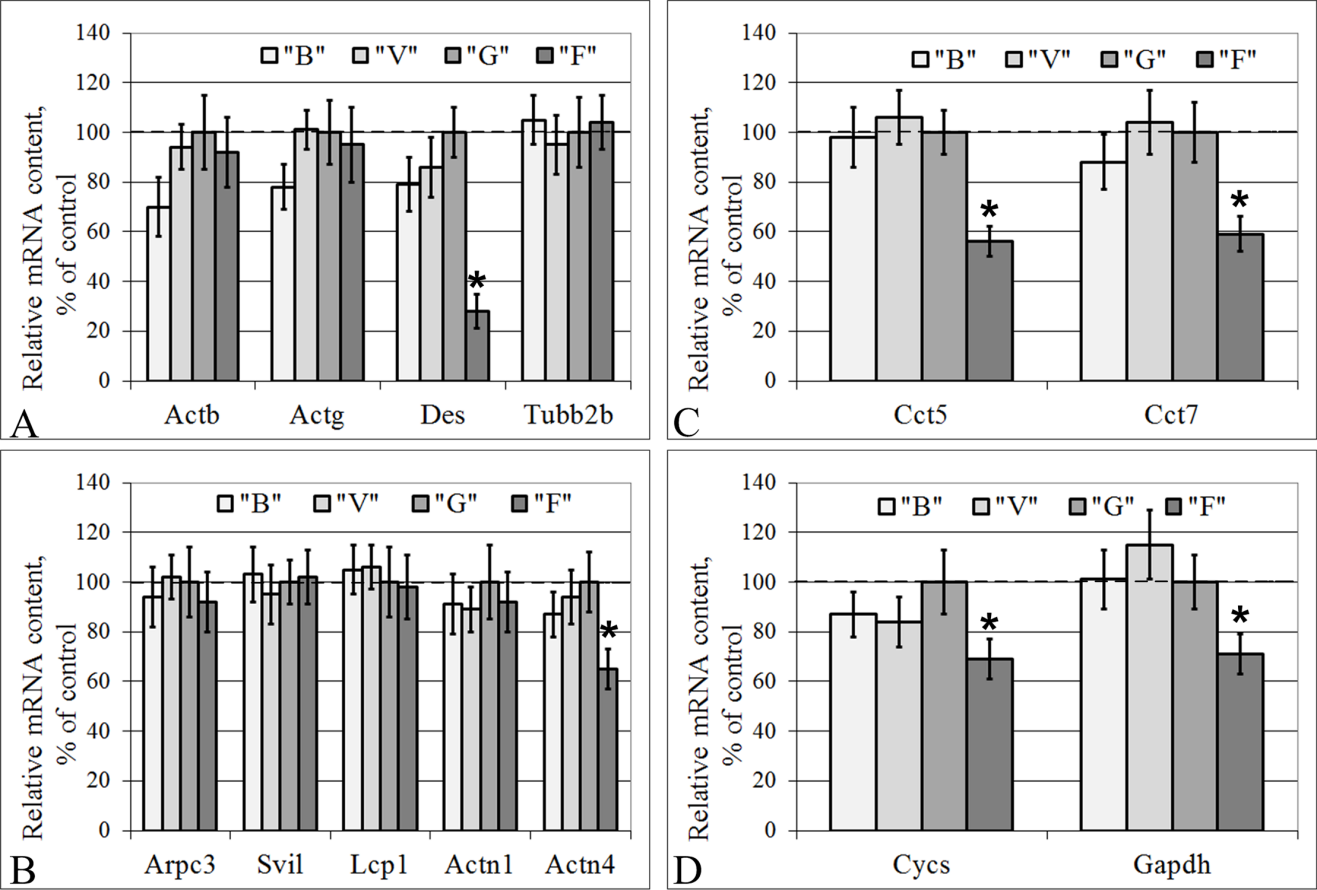
Relative mRNA contents of genes (qPCR data) that encode cytoskeletal and metabolic proteins in the cardiac tissue. (A) The actin isoforms, the microfilaments component; desmin, the intermediate filaments component; subunit 2B of beta-tubulin, the microtubules component. (B) The actin-binding proteins. (C) The tubulin-binding proteins. (D) The metabolic proteins, cytochrome *c* and glyceraldehyde 3-phosphate dehydrogenase. “B”–basal control group, “V”–vivarium control group, “G”–ground control group (level marked by dotted line), “F”–flight group. *–p < 0.05 in comparison with group “G”. The values used to build graphs represented in the S3 Table.

**Fig 4:**
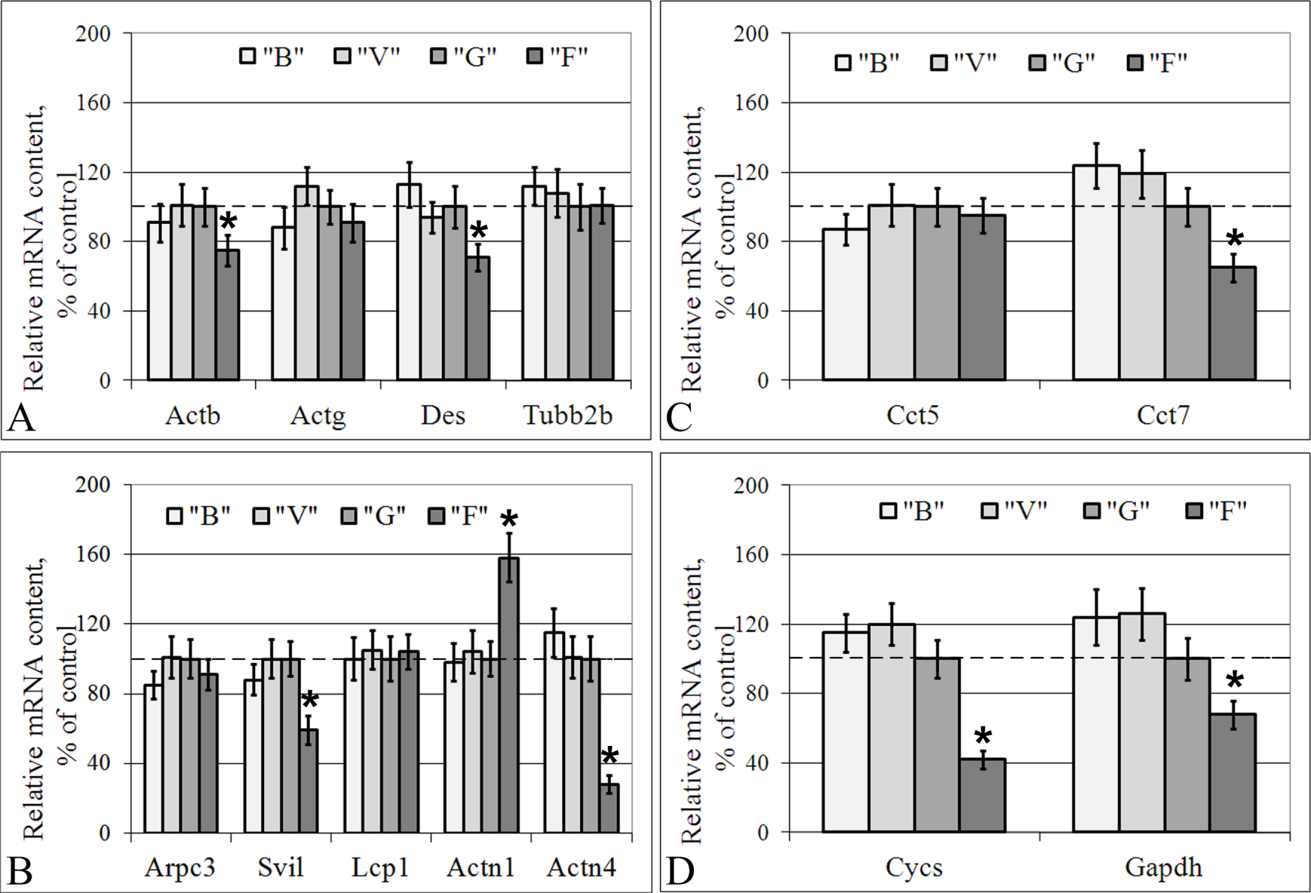
Relative mRNA content of genes (qPCR data) that encode cytoskeletal and metabolic proteins in the lung cells. (A) The actin isoforms, the microfilaments component; desmin, the intermediate filaments component; subunit 2B of beta-tubulin, the microtubules component. (B) The actin-binding proteins. (C) The tubulin-binding proteins. (D) The metabolic proteins, cytochrome *c* and glyceraldehyde 3-phosphate dehydrogenase. “B”–basal control group, “V”–vivarium control group, “G”–ground control group (level marked by dotted line), “F”–flight group. *–p < 0.05 in comparison with group “G”. The values used to build graphs represented in the S4 Table.

In groups B and V, none of the mRNA contents of the examined genes differed from those in group G in either the cells of the left ventricle of the heart or the lung cells (Fig 3 and Fig 4).

The relative mRNA contents of genes encoding beta- and gamma-actin (Fig 3A), certain actin-binding proteins (Arpc3, Svil, Lcp1, Actn1) (Fig 3B), and beta-tubulin (Fig 3A) in the cardiac tissue were similar in all the study groups. The mRNA contents of Des (Fig 3A), Actn4 (Fig 3B), Cct5, Cct7 (Fig 3C), Cycs, and Gapdh (Fig 3D) in group F were reduced compared to those in group G by 72% (p < 0.05), 35% (p < 0.05), 44% (p < 0.05), 41% (p < 0.05), 31% (p < 0.05), and 29% (p < 0.05), respectively.

In the lung tissue, the relative mRNA contents of group F compared to that of group G decreased by 25% for Actb (p < 0.05), 29% for Des (p < 0.05) (Fig 4A), 41% for Svil (p < 0.05), 72% for Actn4 (p < 0.05) (Fig 4B), 35% for Cct7 (p < 0.05) (Fig 4C), 58% for Cycs (p < 0.05), and 32% for Gapdh (p < 0.05) (Fig 4D). The Actn1 mRNA content of group F increased by 58% compared to that of group G (p < 0.05) (Fig 4B). The relative mRNA contents of the Actg, Arpc3, Lcp1, and BetaTub genes were unchanged in group F compared to group G (Fig 4A and 4B).

### DNA total methylation level

**Fig 5:**
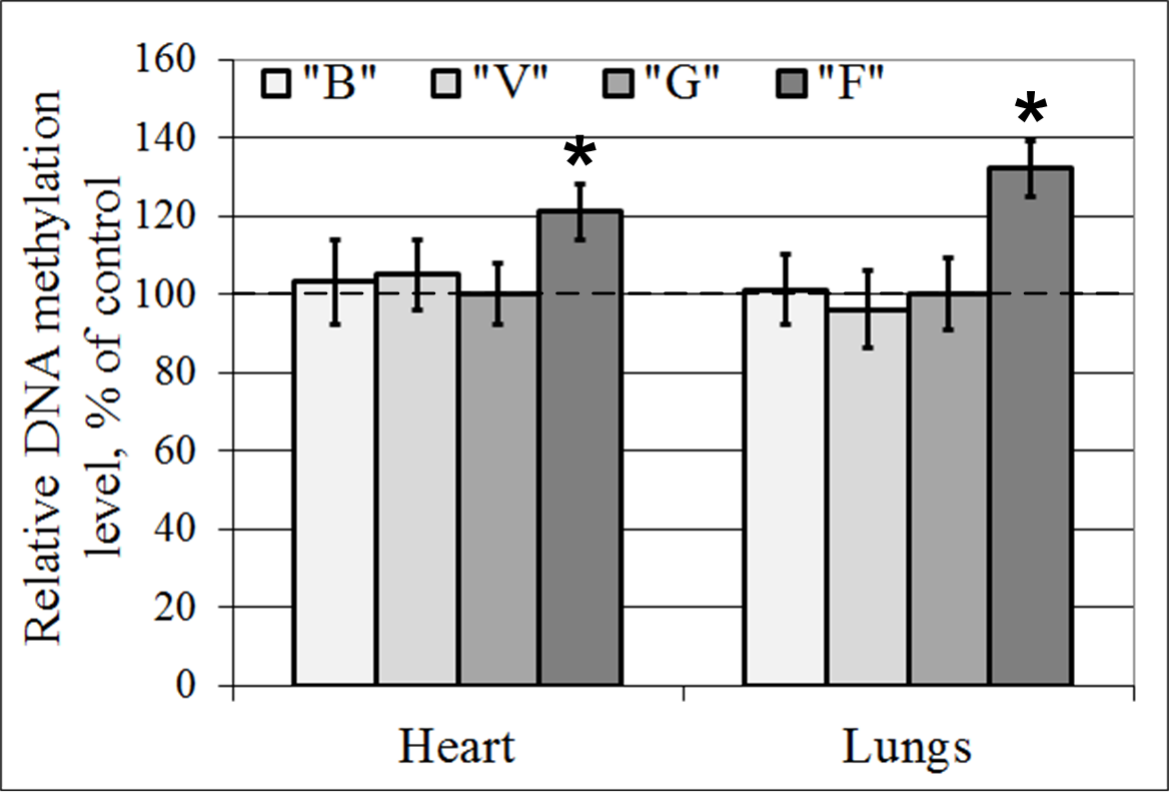
DNA total methylation level following digestion with Epi MspI/Epi HpaII. “B”–basal control group, “V”–vivarium control group, “G”–ground control group, “F”–flight group. *–p < 0.05 in comparison with group “G”. The values used to build graphs represented in the S5 Table.

**Fig 6:**
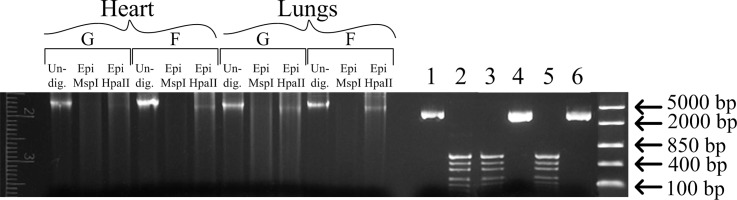
Representative image of the digestion of genomic DNA by Epi MspI and Epi HpaII. Undig.–undigested DNA. Epi MspI–digested with Epi MspI. Epi HpaII–digested with Epi HpaII. 1, 2, 3 –control pUC19/Smal unmethylated (undigested DNA, Epi MspI, and Epi HpaII, respectively). 4, 5, 6 –control mpUC19/Smal methylated (undigested DNA, Epi MspI, and Epi HpaII, respectively). “G”–ground control group (level marked by dotted line), “F”–flight group.

The level of total DNA methylation in groups B and V did not differ from that in group G in either the cardiac or the lung tissue. In the flight group, F, the level of total DNA methylation was higher than that in the control group G by 21% (p < 0.05) in the cardiac tissue and by 32% (p < 0.05) in the lung tissue (Figs 5 and 6). In addition, the major changes in the methylation levels were associated with the methylation of the internal cytosine at the 5’-CCGG-3’ locus, and thus, no changes in the efficiency of MspI (which is blocked by methylation of exogenous cytosine) were observed in any of the study groups (Fig 6).

### The mRNA contents of genes that encode transcriptional regulators: Methylases, demethylases, histone acetylase and histone deacetylase

**Fig 7:**
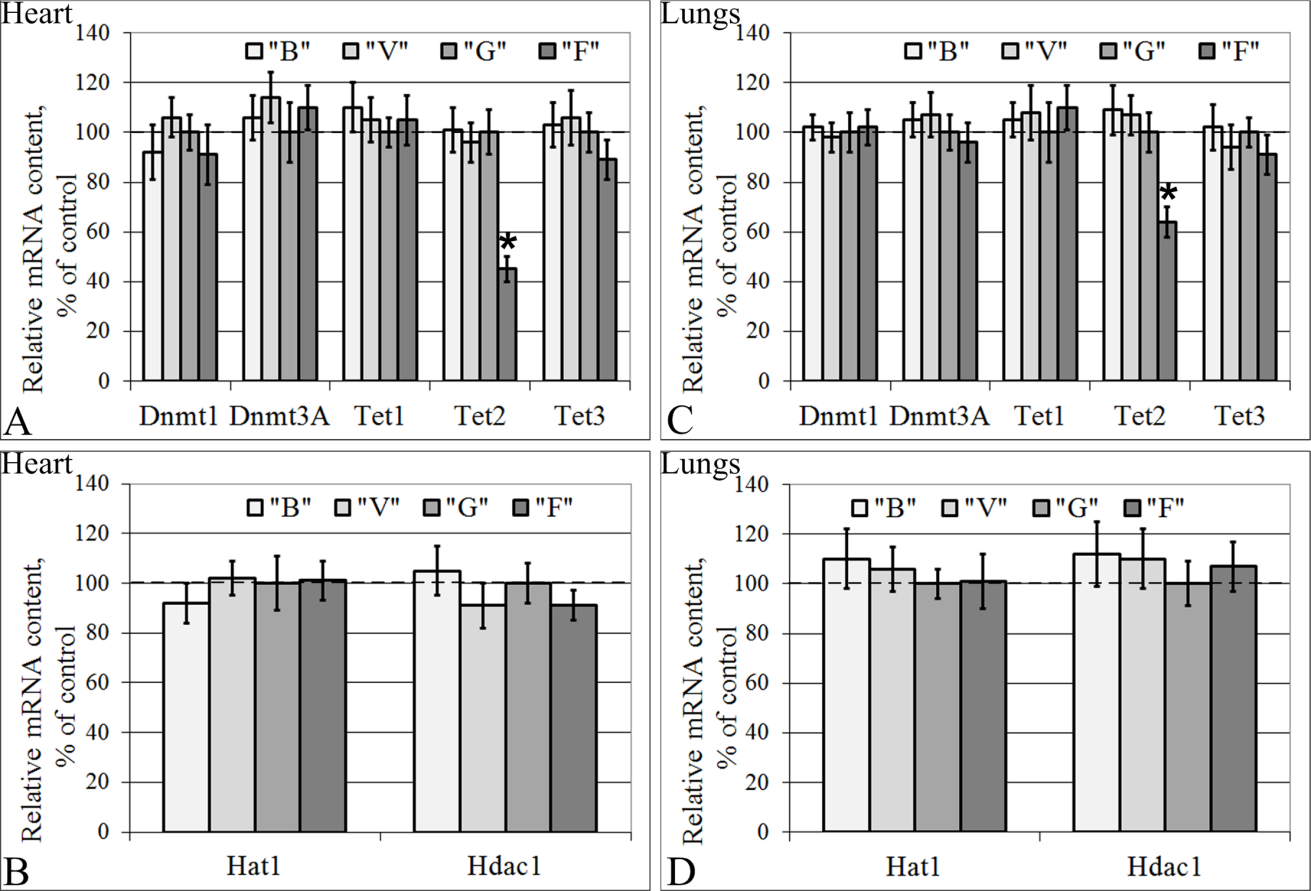
Relative mRNA contents of genes (qPCR data) that encode some regulators of transcription. (A) Heart: the cytosine methylases Dnmt1 (S-phase methylation) and Dnmt3A (*de novo* methylation) and the cytosine demethylases (Tet1, Tet2, Tet3). (B) Heart: the histone aminotransferase 1 Hat1 and Hdac1 (histone deacetylase 1, 2, 3, 4, 6, 9). (C) Lungs: Dnmt1 and Dnmt3A and Tet1, Tet2 and Tet3. (D) Lungs: Hat1 and Hdac1. “B”–basal control group, “V”–vivarium control group, “G”–ground control group (level marked by dotted line), “F”–flight group. *–p < 0.05 in comparison with group “G”. The values used to build graphs represented in the S6 and S7 Tables.

The mRNA contents of genes encoding proteins involved in transcriptional regulation in groups B and V did not differ from those in group G in either the cardiac or lung tissue (Fig 7). Moreover, in the cells of both tissue types in the flight group, F, the relative mRNA contents of genes encoding the methylases Dnmt1 (S-phase methylation) and Dnmt3A (*de novo* methylation), the cytosine demethylases Tet1 and Tet3 (Fig 7A and 7C), the histone aminotransferase Hat1 and the histone deacetylase Hdac1 (Fig 7B and 7D) did not differ from those of the control group. However, the mRNA content of the cytosine demethylase Tet2 decreased by 55% (p < 0.05) in the cardiac tissue (Fig 7A) and 36% (p < 0.05) in the lung tissue (Fig 7C) in the flight group compared to the control group.

## Discussion

The influence of the changes in external mechanical conditions, particularly gravity changes, on the cells of organisms that have evolved in Earth’s gravity conditions is still poorly understood. The main difficulty is the extrapolation of ground experimental data to the prediction of results under weightlessness conditions. Previous experiments conducted under space flight conditions were limited by the fact that the biomaterial of the flight animals could only be fixed after landing, usually occurring a few hours after the landing [1–7, 13]. Therefore, this experiment is the first in the history of human space exploration with the unique opportunity to fix mouse biomaterial under space flight conditions. Comparing these results with the results obtained after the BION-M Biosatellite No. 1 flight of the same length, a number of deductions can be made regarding the structural changes in the cells as a result of the early period of readaptation to Earth’s gravity.

### Long duration microgravity does not lead to notable changes in cytoskeletal protein contents in the cardiac and lung tissue

The relative contents of beta-actin, gamma-actin, beta-tubulin and desmin both in membrane and cytoplasmic fractions of the cardiac tissue remained at the same level in all the study groups. These data agree with data Tauber S. et al. showed for macrophages, which were also recorded under weightlessness conditions in the ISS (Space-X3 mission). In addition, changes of the cytoskeleton structure after an 11-day spaceflight were not observed [23].

The contents of the actin-binding proteins alpha-actinin-1 and alpha-actinin-4 in the membrane fraction were also unchanged. However, in the cytoplasmic fraction, alpha-actinin-1 decreased by 40% (p < 0.05), and alpha-actinin-4 decreased by 30% (p < 0.05) in group F compared to group G.

The previous data from the 30-day space flight of the BION-M Biosatellite No. 1 (2013, Russia) showed that the beta-actin content in the membrane fraction of proteins was at the same level, while in the cytoplasmic fraction, there was a decrease in the beta-actin protein and mRNA expression levels in the flight group [13]. The gamma-actin mRNA and protein contents were unchanged. The alpha-actinin-1 protein content remained unchanged in the post-flight group, while the corresponding mRNA content was higher in the post-flight group than in the control groups. The alpha-actinin-4 protein content in the membrane fraction was reduced by 28% (p < 0.05) in group F compared to the control, although there was not a similar change in the cytoplasmic fraction. At the same time, the corresponding gene expression was reduced by 18% [13]. The principal difference between the RR-1 and BION-M No. 1 experiments is that the biomaterial in the RR-1 experiment was fixed under space flight conditions, and the biomaterial in the BION-M Biosatellite No. 1 experiment was fixed within 13 hours after landing, i.e., there was a brief period of readaptation. In addition, another critical point is the effects of congestion during landing. However, overload during early readaptation is also a sharp increase of the external mechanical load.

Earlier, we proposed a model wherein changes of the external mechanical load can lead to the dissociation of actin-binding proteins from the cortical cytoskeleton and the triggering signaling pathways, leading to a change in the expression level of the corresponding genes, which in turn leads to a redistribution of cytoskeletal proteins between the cellular compartments [38]. Comparison of the data obtained from the RR-1 experiment with the data of the BION-M biosatellite experiment (Table 2) suggests that long-term space flight leads to a decrease in the expression levels of alpha-actinin-1 and alpha-actinin-4 in the cytoplasmic fraction of cardiac proteins. Thirteen hours under Earth’s gravity after a long-term space flight causes a reduction in the beta-actin content in the cytoplasmic fraction of proteins, the recovery of the alpha-actinin-1 content to the reference level and the reallocation of alpha-actinin-4 between the membrane and cytoplasmic fractions.

**Table 2:** Comparison of the RR-1 experiment with BION-M No.1 experiment (for cardiac tissue).

Parameter	RR-1	BION-M No.1
Duration of flight	37 days	30 days
Period of early readaptation	none	13 hours
ACTB relative content, MF/CF/mRNA	~/~/~	~/↓/↓
ACTG relative content, MF/CF/mRNA	~/~/~	~/~/~
ACTN1 relative content, MF/CF/mRNA	~/↓/~	~/~/↑
ACTN4 relative content, MF/CF/mRNA	~/↓/↓	↓/~/↓

ACTB–beta-actin, ACTG–gamma-actin, ACTN1 –alpha-actinin-1, ACTN4 –alpha-actinin-4, CF–cytoplasmic fraction, MF–membrane fraction.

Thus, it seems that a long-duration space flight leads to the adaptation of the cortical cytoskeleton protein pattern to the weightlessness conditions. Cytoskeleton changes, observed in the early period of readaptation, can subsequently affect myocardial function. Summarizing these data, it can be assumed that the early period of readaptation is more damaging than space flight. Therefore, we can suggest that the development of protective measures could possibly focus on preparing for landing.

It should be noted that only minor changes in the protein contents in the cytoplasmic fraction of the cardiac tissue were found, and no changes were detected in the lung tissue. Therefore, this finding raises the question of whether the absence of changes in the protein contents was associated with the absence of changes in gene expression and/or changes in translation/proteolysis efficiency. Thus, qRT-PCR was performed to detect the mRNA content.

For the cardiac tissue, we found no changes in the mRNA contents of genes encoding the isoforms of actin and tubulin (the protein contents were also unchanged). The mRNA content of the alpha-actinin-4 gene was reduced, as was the protein content. At the same time, in the cardiac and lung tissue, there was a decrease of the desmin mRNA content with an absence of the protein content. In addition to the desmin mRNA changes in the lung tissue with an absence of consequent protein content changes, the beta-actin mRNA content was also decreased. This finding suggests an increase of translation efficiency and/or decrease in the efficiency of the proteolysis of these proteins.

One possible method is the release of phospholipase D, which leads to an increase in translation efficiency. In turn, phospholipase D associates with alpha-actinin-1, which inhibits their activity [39]. Accordingly, if the formation of the protein pattern in the weightlessness (after short-term duration) passed the stage of the cytoskeleton reorganization (it can be assumed on the basis of a number of model experiments [40, 41, 42]), phospholipase D could be released, leading to the formation of the observed protein pattern after long-term duration in the microgravity conditions.

However, in the absence of changes in the alpha-actinin-1 mRNA content, the protein content in the cytoplasm of cardiomyocytes decreased; in the lung tissue, the alpha-actinin-1 protein content remained at control levels, but the mRNA content increased. Alpha-actinin-1 is a member of the spectrin family [43], which is located along stress-fibrils and connects microfilament bundles [44]. Alpha-actinin-1 (as well as alpha-actinin-4) is a non-muscle isoform of the alpha-actinins, but it is expressed in cardiomyocytes [45] and is able to interact with a large number of signaling molecules and transcriptional factors [46, 47]. The reduction of the alpha-actinin-1 content may occur in the cytoplasmic fraction as a result of its migration into the nucleus. Although we did not detect alpha-actinin-1 in the nucleus, its highly homologous protein alpha-actinin-4 can transfer to the nucleus and bind to the promoter regions of certain genes [48].

It should be mentioned that procedure of freeze-defrost-freeze for the estimated tissues could lead to decreased protein content. However, we estimated the relative contents of the proteins and mRNA. Because we compared the flight group results with those of the control groups and the procedure for all groups was identical, we can estimate relative changes in the flight group.

### Total transcriptional efficiency of DNA of mouse cardiac and lung tissue after a long duration space flight

The cause of the reduction of the mRNA content of certain genes that encode cytoskeletal proteins remains unclear. The transcription efficiency of eukaryotic promoters, especially in higher animals, is closely related to the level of genome methylation. DNA from mammalian somatic tissue is methylated in 70% of all CpG sites [49]. The key exceptions to this global methylation are the CpG islands, which mark the promoters and 5’- domains of genes [50]. The reported data regarding DNA methylation in rice plants, human T-lymphocyte cells and human lymphoblastoid cells under space flight conditions and the results of modeling experiments suggest that DNA methylation patterns change [32, 33, 34]. Therefore, we decided to estimate the total methylation level in our samples. We showed that methylation in the mouse cardiac and lung tissue shifted to hypermethylation after a 37-day space flight. In addition, these data agreed with the data that Ou X. et al. [32] obtained after space flight.

However, the mechanisms underlying the changes of the methylation levels after space flight are currently unknown. The methylation level of the genome increased because heavy ions radiation, which can make a difference in the space flight conditions [51, 52]. In addition, this can contribute to the observed increase in the methylation level in the flight group. However, this contribution is unlikely to be decisive, since this experiment was conducted on board an International Space Station. Animals and humans were in the same compartments, which have both special protection against cosmic radiation and natural protection, since the orbit passes below the Earth's magnetosphere.

In general, it is known that that *de novo* methylation can be mediated by histone deacetylation in the nucleosome area [53–58]. In addition, changes of methylation levels can result from increases in the methylase activity/content. Singh K.P. et al. [33] showed that the expression levels of DNMT1, DNMT3a, and DNMT3b were increased at 72 h and decreased at 7 days in microgravity-exposed cells, but at the same time, there was decreased expression of HDAC1. The authors suggested that the decreased expression of HDAC1 would result in an increased level of acetylated histone H3; however, a decreased level of acetylated H3 was observed under the microgravity condition, which indicated that other HDACs may be involved in the regulation of H3 deacetylation [33]. Ou X. et al. [32] found that several genes encoding the various putative DNA methyltransferases, 5-methylcytosine DNA glycosylases, the SWI/SNF chromatin remodeler (DDM1) and siRNA-related proteins are extremely sensitive to perturbation by space flight, which may be an underlying cause for the altered methylation patterns in plants subjected to space flight.

Therefore, we decided to assess the expression level of genes encoding methylases as well as the basic histone acetylase/deacetylase. The obtained results indicate that the gene expression levels of the histone aminotransferase Hat1, the histone deacetylase Hdac1, and the cytosine methylases Dnmt1 (S-phase methylation) and Dnmt3A (*de novo* methylation) were unchanged under weightlessness conditions.

On the other hand, the methylation level can increase as a result of a reduction of the intensity of active DNA demethylation. Researchers believe that 5 hmC is the intermediate product in the process of active DNA demethylation [59–63] and is generated by Ten-Eleven Translocation (TET) protein-mediated oxidative catalysis of 5mC [64].

Therefore, we measured the mRNA content of cytosine demethylases (Tet1, Tet2, Tet3) in the tested tissues. There were no changes in Tet1 and Tet3 mRNA content, but there was a significant decrease Tet2 mRNA content in mouse cardiac and lungs tissues. The data regarding Tet2 contents are quite limited and inconsistent; in TET2-mutated acute myeloid leukemia samples, Ko M. et al. [65] observed DNA hypomethylation, but Figueroa M.E. et al. [66] showed a DNA hypermethylation phenotype. Moreover, Menon M.P. et al. [67] showed that patients with primary central nervous system T-cell lymphomas had tumor cell necrosis as well as mutations of a number of genes, in particular TET2, the products of which are involved in the regulation of transcription efficiency. Thus, in our experiment, we can only assume that the mechanism of the change in total methylation levels in the cardiac and lung tissue of mice was associated with the decrease in the Tet2 mRNA content under space flight conditions.

## Conclusions

Our study, in which murine biomaterial was fixed for the first time under space flight conditions (after 37 days of weightlessness), showed no changes in the cytoskeletal protein contents in the cardiac and lung tissue of mice. At the same time, there were significant changes in the mRNA content, which, on the one hand, indicates changes in translation efficiency and/or proteolysis levels that act to maintain normal levels of proteins and on the other hand, indicates the alteration of the expression levels of genes encoding cytoskeletal proteins, perhaps as a result of the total genome methylation increase. The mRNA contents of the DNA methylases, the cytosine demethylases Tet1 and Tet3, histone acetylase and histone deacetylase were unchanged, and the mRNA content of the cytosine demethylase Tet2 was significantly decreased, which could cause changes in the total methylation level.

## Supporting information

S1 Table Relative contents of cytoskeletal proteins (% of control) in the membrane (MF) and cytoplasmic (CF) fractions of cardiomyocytes. “B”–basal control group, “V”–vivarium control group, “G”–ground control group, “F”–flight group. CF–cytoplasmic fraction, MF–membrane fraction. *–p < 0.05 in comparison with group “G”.
(DOCX)

S2 Table Relative contents of cytoskeletal proteins (% of control) in the membrane (MF) and cytoplasmic (CF) fractions of lung cells. “B”–basal control group, “V”–vivarium control group, “G”–ground control group, “F”–flight group.
(DOCX)

S3 Table Relative mRNA contents (% of control) of genes (qPCR data) that encode cytoskeletal and metabolic proteins in the cardiac tissue. “B”–basal control group, “V”–vivarium control group, “G”–ground control group, “F”–flight group. *–p < 0.05 in comparison with group “G”.
(DOCX)

S4 Table Relative mRNA contents (% of control) of genes (qPCR data) that encode cytoskeletal and metabolic proteins in the lung cells. “B”–basal control group, “V”–vivarium control group, “G”–ground control group, “F”–flight group. *–p < 0.05 in comparison with group “G”.
(DOCX)

S5 Table DNA total methylation level (% of control) following digestion with Epi MspI/Epi HpaII. “B”–basal control group, “V”–vivarium control group, “G”–ground control group, “F”–flight group. *–p < 0.05 in comparison with group “G”.
(DOCX)

S6 Table Relative mRNA contents (% of control) of genes (qPCR data) that encode some regulators of transcription in the heart tissue. “B”–basal control group, “V”–vivarium control group, “G”–ground control group, “F”–flight group. *–p < 0.05 in comparison with group “G”.
(DOCX)

S7 Table Relative mRNA contents (% of control) of genes (qPCR data) that encode some regulators of transcription in the lung tissue. “B”–basal control group, “V”–vivarium control group, “G”–ground control group, “F”–flight group. *–p < 0.05 in comparison with group “G”.
(DOCX)

S8 Table The ARRIVE guidelines checklist. Animal Research: Reporting In Vivo Experiments.
(DOCX)
