# Supplementary material for: Cytoskeleton structure and total methylation of mouse cardiac and lung tissue during space flight
Source: PLoS One. 2018 May 16;13(5):e0192643. doi: 10.1371/journal.pone.0192643 (PMC5955502; doi:10.1371/journal.pone.0192643)
Supplement: S1 Table — “B”–basal control group, “V”–vivarium control group, “G”–ground control group, “F”–flight group. CF–cytoplasmic fraction, MF–membrane fraction. *–p < 0.05 in comparison with group “G”. (DOCX) [file pone.0192643.s001.docx]

**S1 Table. Relative contents of cytoskeletal proteins (% of control) in the membrane (MF) and cytoplasmic (CF) fractions of cardiomyocytes.**

| Protein | Fraction | B | V | G | F |
| --- | --- | --- | --- | --- | --- |
| Alpha-actinin-1 | MF | 88 ± 9 | 92 ± 11 | 100 ± 10 | 94 ± 10 |
|  | CF | 91 ± 9 | 108 ± 12 | 100 ± 11 | 60 ± 7* |
| Alpha-actinin-4 | MF | 105 ± 12 | 108 ± 10 | 100 ± 11 | 89 ± 11 |
|  | CF | 110 ± 11 | 94 ± 9 | 100 ± 10 | 70 ± 8* |
| Beta-actin | MF | 89 ± 11 | 112 ± 13 | 100 ± 12 | 89 ± 11 |
|  | CF | 104 ± 12 | 89 ± 13 | 100 ± 11 | 110 ± 9 |
| Gamma-actin | MF | 94 ± 9 | 89 ± 12 | 100 ± 12 | 94 ± 10 |
|  | CF | 105 ± 10 | 94 ± 11 | 100 ± 13 | 108 ± 12 |
| Beta-tubulin | MF | 98 ± 10 | 95 ± 11 | 100 ± 12 | 95 ± 9 |
|  | CF | 112 ± 12 | 108 ± 11 | 100 ± 10 | 114 ± 11 |
| Desmin |  | 94 ± 9 | 89 ± 12 | 100 ± 12 | 94 ± 10 |

“B” – basal control group, “V” – vivarium control group, “G” – ground control group, “F” – flight group. CF – cytoplasmic fraction, MF – membrane fraction. * – p < 0.05 in comparison with group “G”.
