# Supplementary material for: Cytoskeleton structure and total methylation of mouse cardiac and lung tissue during space flight
Source: PLoS One. 2018 May 16;13(5):e0192643. doi: 10.1371/journal.pone.0192643 (PMC5955502; doi:10.1371/journal.pone.0192643)
Supplement: S2 Table — “B”–basal control group, “V”–vivarium control group, “G”–ground control group, “F”–flight group. (DOCX) [file pone.0192643.s002.docx]

**S2 Table. Relative contents of cytoskeletal proteins (% of control) in the membrane (MF) and cytoplasmic (CF) fractions of lung cells.**

| Protein | Fraction | B | V | G | F |
| --- | --- | --- | --- | --- | --- |
| Alpha-actinin-1 | MF | - | - | 100 ± 14 | 109 ± 12 |
|  | CF | 104 ± 12 | 107 ± 11 | 100 ± 9 | 113 ± 11 |
| Alpha-actinin-4 | MF | - | - | 100 ± 13 | 112 ± 12 |
|  | CF | 98 ± 13 | 88 ± 11 | 100 ± 12 | 97 ± 12 |
| Beta-actin | MF | - | - | 100 ± 11 | 102 ± 13 |
|  | CF | 89 ± 11 | 88 ± 10 | 100 ± 13 | 94 ± 12 |
| Gamma-actin | MF | - | - | 100 ± 11 | 87 ± 12 |
|  | CF | 84 ± 12 | 112 ± 16 | 100 ± 11 | 109 ± 12 |
| Beta-tubulin | MF | - | - | 100 ± 11 | 86 ± 10 |
|  | CF | 104 ± 11 | 114 ± 13 | 100 ± 12 | 110 ± 12 |
| Desmin |  | 110 ± 11 | 94 ± 12 | 100 ± 10 | 106 ± 12 |

“B” – basal control group, “V” – vivarium control group, “G” – ground control group, “F” – flight group.
