# Supplementary material for: Cytoskeleton structure and total methylation of mouse cardiac and lung tissue during space flight
Source: PLoS One. 2018 May 16;13(5):e0192643. doi: 10.1371/journal.pone.0192643 (PMC5955502; doi:10.1371/journal.pone.0192643)
Supplement: S3 Table — “B”–basal control group, “V”–vivarium control group, “G”–ground control group, “F”–flight group. *–p < 0.05 in comparison with group “G”. (DOCX) [file pone.0192643.s003.docx]

**S3 Table. Relative mRNA contents (% of control) of genes (qPCR data) that encode cytoskeletal and metabolic proteins in the cardiac tissue.**

| Gene | B | V | G | F |
| --- | --- | --- | --- | --- |
| *Actb* (beta-actin) | 70 ± 12 | 94 ± 9 | 100 ± 15 | 92 ± 14 |
| *Actg* (gamma-actin) | 78 ± 9 | 101 ± 8 | 100 ± 13 | 95 ± 15 |
| *Des* (desmin) | 79 ± 11 | 86 ± 12 | 100 ± 10 | 28 ± 7* |
| *Tubb2b* (beta-tubulin 2B) | 105 ± 10 | 95 ± 12 | 100 ± 14 | 104 ± 11 |
| *Arpc3* (actin-related protein 2/3 complex, subunit 3) | 94 ± 12 | 102 ± 9 | 100 ± 14 | 92 ± 12 |
| *Svil* (supervillin) | 103 ± 11 | 95 ± 12 | 100 ± 9 | 102 ± 11 |
| *Lcp1* (L-plastin) | 105 ± 10 | 106 ± 9 | 100 ± 14 | 98 ± 13 |
| *Actn1* (alpha-actinin 1) | 91 ± 12 | 89 ± 9 | 100 ± 15 | 92 ± 12 |
| *Actn4* (alpha-actinin 4) | 87 ± 9 | 94 ± 11 | 100 ± 12 | 65 ± 8* |
| *Cct5* (chaperonin containing Tcp-1, subunit 5 - epsilon) | 98 ± 12 | 106 ± 11 | 100 ± 9 | 56 ± 6* |
| *Cct7* (chaperonin containing Tcp-1, subunit 7 - eta) | 88 ± 11 | 104 ± 13 | 100 ± 12 | 59 ± 7* |
| *Cycs* (cytochrome *c*) | 87 ± 9 | 84 ± 10 | 100 ± 13 | 69 ± 8* |
| *Gapdh* (glyceraldehyde 3-phosphate dehydrogenase) | 101 ± 12 | 115 ± 14 | 100 ± 11 | 71 ± 8* |

“B” – basal control group, “V” – vivarium control group, “G” – ground control group, “F” – flight group. * – p < 0.05 in comparison with group “G”.
