# Supplementary material for: Cytoskeleton structure and total methylation of mouse cardiac and lung tissue during space flight
Source: PLoS One. 2018 May 16;13(5):e0192643. doi: 10.1371/journal.pone.0192643 (PMC5955502; doi:10.1371/journal.pone.0192643)
Supplement: S5 Table — “B”–basal control group, “V”–vivarium control group, “G”–ground control group, “F”–flight group. *–p < 0.05 in comparison with group “G”. (DOCX) [file pone.0192643.s005.docx]

**S5 Table. DNA total methylation level (% of control) following digestion with Epi MspI/Epi HpaII.**

| Tissue | B | V | G | F |
| --- | --- | --- | --- | --- |
| Heart | 103 ± 11 | 105 ± 9 | 100 ± 8 | 121 ± 7* |
| Lungs | 101 ± 9 | 96 ± 10 | 100 ± 9 | 132 ± 7* |

“B” – basal control group, “V” – vivarium control group, “G” – ground control group, “F” – flight group. * – p < 0.05 in comparison with group “G”.
