# Supplementary material for: Cytoskeleton structure and total methylation of mouse cardiac and lung tissue during space flight
Source: PLoS One. 2018 May 16;13(5):e0192643. doi: 10.1371/journal.pone.0192643 (PMC5955502; doi:10.1371/journal.pone.0192643)
Supplement: S6 Table — “B”–basal control group, “V”–vivarium control group, “G”–ground control group, “F”–flight group. *–p < 0.05 in comparison with group “G”. (DOCX) [file pone.0192643.s006.docx]

**S6 Table. Relative mRNA contents (% of control) of genes (qPCR data) that encode some regulators of transcription in the heart tissue.**

| Gene | B | V | G | F |
| --- | --- | --- | --- | --- |
| *Dnmt1* (S-phase methylation) | 92 ± 11 | 106 ± 8 | 100 ±7 | 91 ± 12 |
| *Dnmt3A* (*de novo* methylation) | 106 ± 9 | 114 ± 10 | 100 ± 12 | 110 ± 9 |
| *Tet1* (cytosine demethylase) | 110 ± 10 | 105 ± 9 | 100 ± 6 | 105 ± 10 |
| *Tet2* (cytosine demethylase) | 101 ± 9 | 96 ± 8 | 100 ± 9 | 45 ± 5* |
| *Tet3* (cytosine demethylase) | 103 ± 9 | 106 ± 11 | 100 ± 8 | 89 ± 8 |
| *Hat1* (histone aminotransferase 1) | 92 ± 8 | 102 ± 7 | 100 ± 11 | 101 ± 8 |
| *Hdac1* (histone deacetylase 1,2,3,4,6,9) | 105 ± 10 | 91 ± 9 | 100 ± 8 | 91 ± 6 |

“B” – basal control group, “V” – vivarium control group, “G” – ground control group, “F” – flight group. * – p < 0.05 in comparison with group “G”.
