# Supplementary material for: Cytoskeleton structure and total methylation of mouse cardiac and lung tissue during space flight
Source: PLoS One. 2018 May 16;13(5):e0192643. doi: 10.1371/journal.pone.0192643 (PMC5955502; doi:10.1371/journal.pone.0192643)
Supplement: S7 Table — “B”–basal control group, “V”–vivarium control group, “G”–ground control group, “F”–flight group. *–p < 0.05 in comparison with group “G”. (DOCX) [file pone.0192643.s007.docx]

**S7 Table. Relative mRNA contents (% of control) of genes (qPCR data) that encode some regulators of transcription in the lung tissue.**

| Gene | B | V | G | F |
| --- | --- | --- | --- | --- |
| *Dnmt1* (S-phase methylation) | 102 ± 5 | 98 ± 6 | 100 ± 8 | 102 ± 7 |
| *Dnmt3A* (*de novo* methylation) | 105 ± 7 | 107 ± 9 | 100 ± 7 | 96 ± 8 |
| *Tet1* (cytosine demethylase) | 105 ± 7 | 108 ± 11 | 100 ± 12 | 110 ± 9 |
| *Tet2* (cytosine demethylase) | 109 ± 10 | 107 ± 8 | 100 ± 8 | 64 ± 6* |
| *Tet3* (cytosine demethylase) | 102 ± 9 | 94 ± 9 | 100 ± 6 | 91 ± 8 |
| *Hat1* (histone aminotransferase 1) | 110 ± 12 | 106 ± 9 | 100 ± 6 | 101 ± 11 |
| *Hdac1* (histone deacetylase 1,2,3,4,6,9) | 112 ± 13 | 110 ± 12 | 100 ± 9 | 107 ± 10 |

“B” – basal control group, “V” – vivarium control group, “G” – ground control group, “F” – flight group. * – p < 0.05 in comparison with group “G”.
