# Supplementary material for: Cytoskeleton structure and total methylation of mouse cardiac and lung tissue during space flight
Source: PLoS One. 2018 May 16;13(5):e0192643. doi: 10.1371/journal.pone.0192643 (PMC5955502; doi:10.1371/journal.pone.0192643)

**S8 Table. The ARRIVE Guidelines Checklist. Animal Research: Reporting In Vivo Experiments**


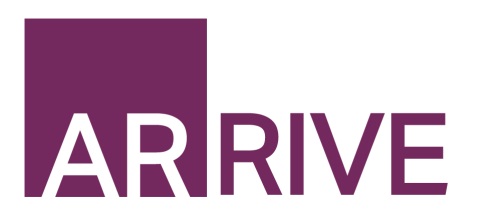


The ARRIVE Guidelines Checklist

Animal Research: Reporting In Vivo Experiments

|  | | ITEM | RECOMMENDATION | Section/ Paragraph |
| --- | --- | --- | --- | --- |
| 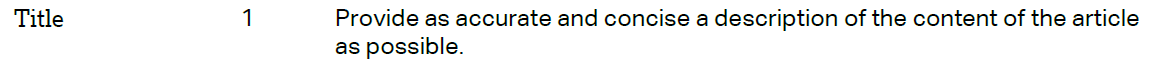 | | | Title |  |
| 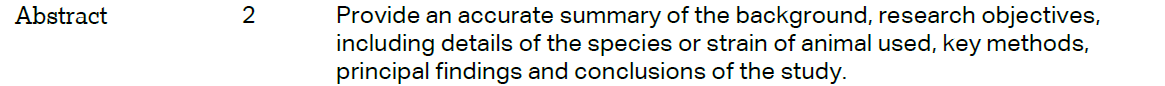 | | | Abstract |  |
| INTRODUCTION | | |  |  |
| 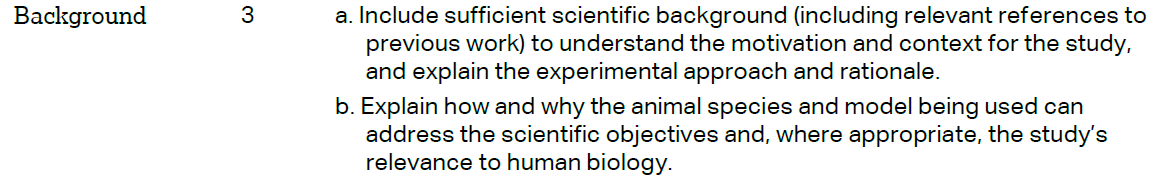 | | | Paragraphs 1-8  Paragraphs 2-3 |  |
| 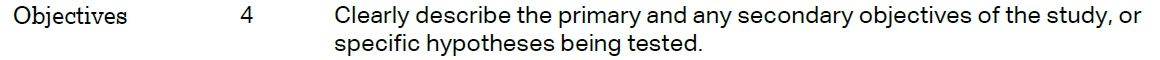 | | | Paragraphs 9-10 |  |
| METHODS | | |  |  |
| 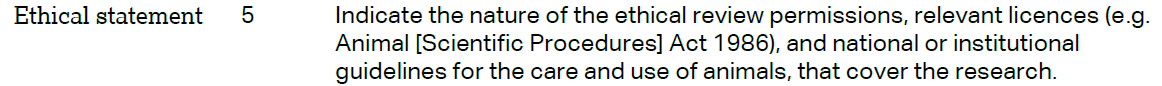 | | | Experimental design  Paragraph 4 |  |
| 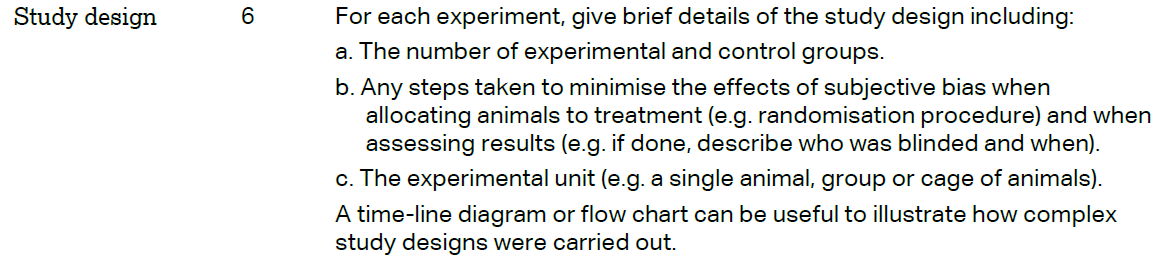 | | | Experimental design  Paragraph 1-2  Paragraph 1-2  Paragraph 3  Paragraph 2-3 |  |
| 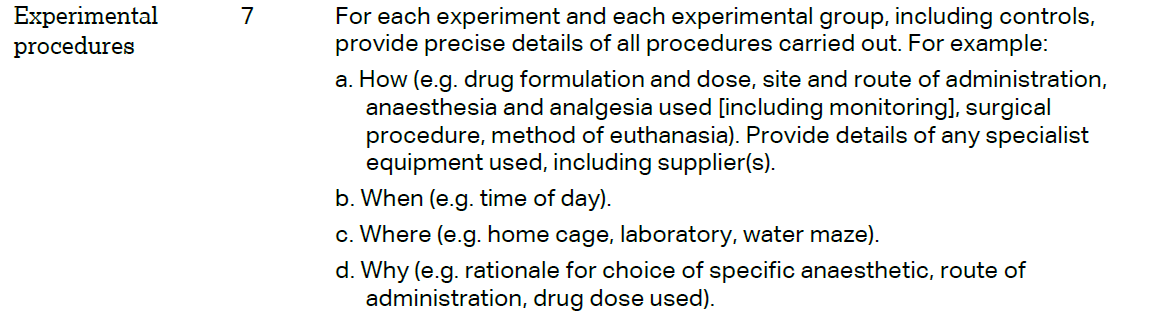 | | | Experimental design  Paragraph 1-5 |  |
| 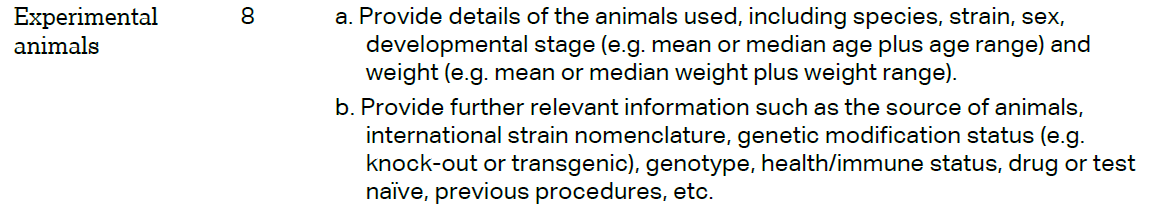 | | | Experimental design  Paragraph 1 |  |

| 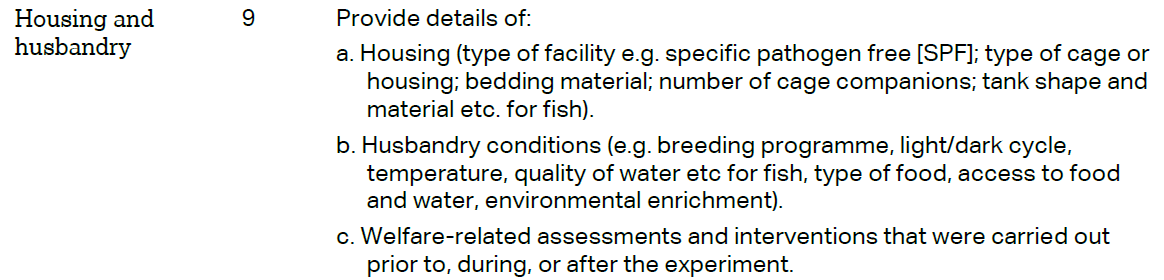 | Experimental design  Paragraph 1-2 | |
| --- | --- | --- |
| 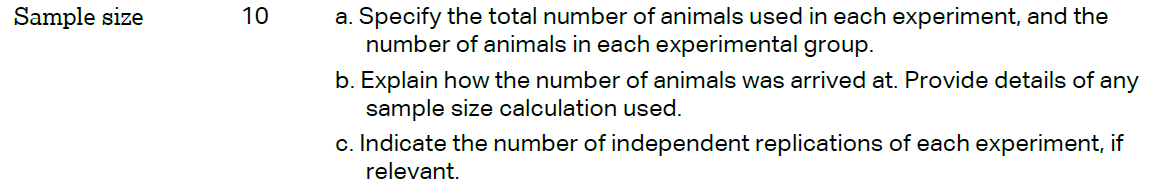 |  | |
| 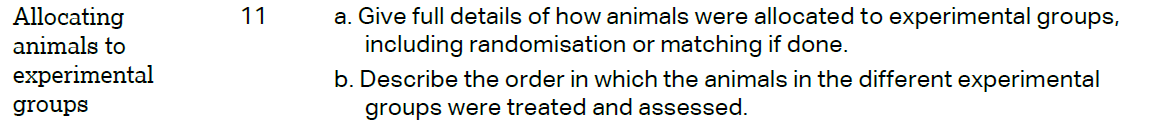 | Experimental design  Paragraph 1-2 | |
| 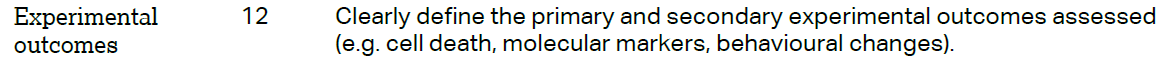 | Paragraph 6-8 | |
| 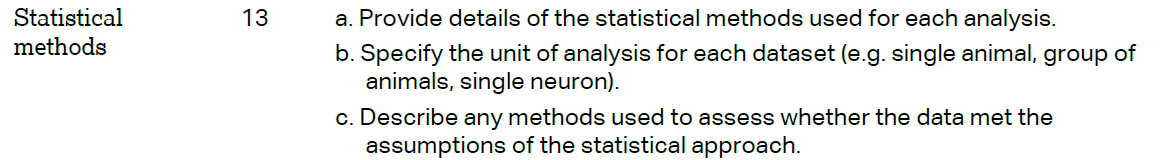 | Paragraph 9 | |
| RESULTS |  | |
| 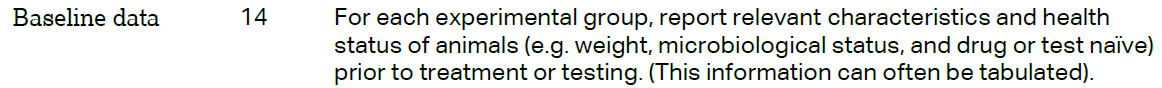 | Methods  Paragraph 1-3 | |
| 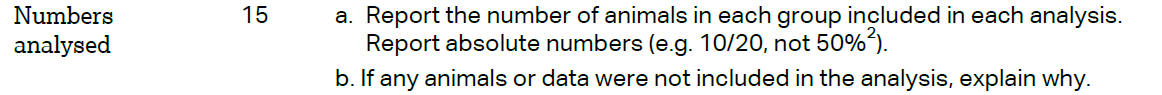 | Methods  Paragraph 3 | |
| 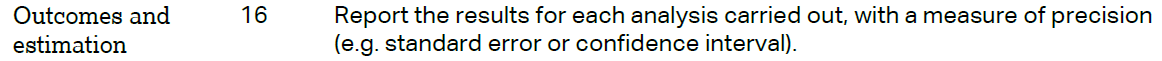 | Paragraphs  1-6 and Figures 1-7 | |
| 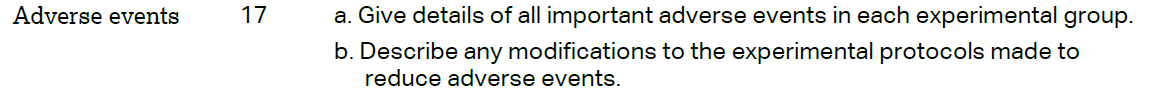 | Paragraph 2 | |
| DISCUSSION |  | |
| 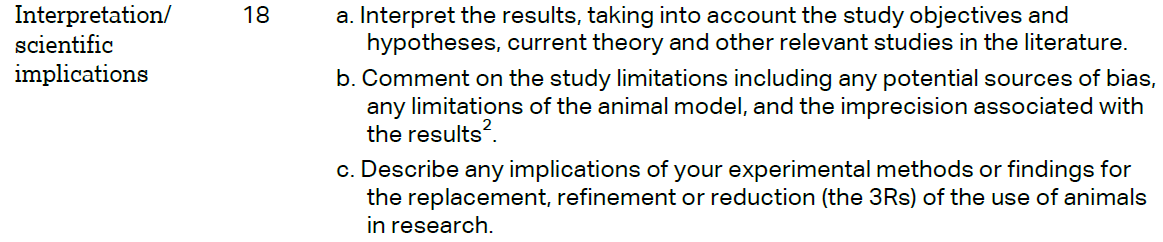 | Throughout | |
| 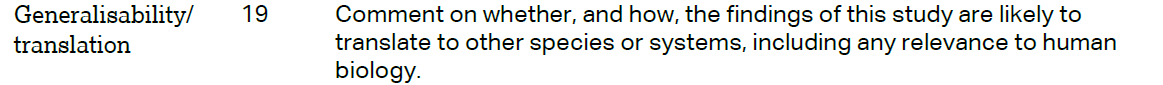 | Introduction  Paragraph 8  Conclusion | |
| 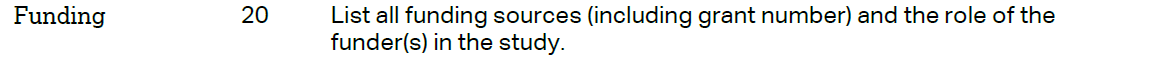 | | Grants |


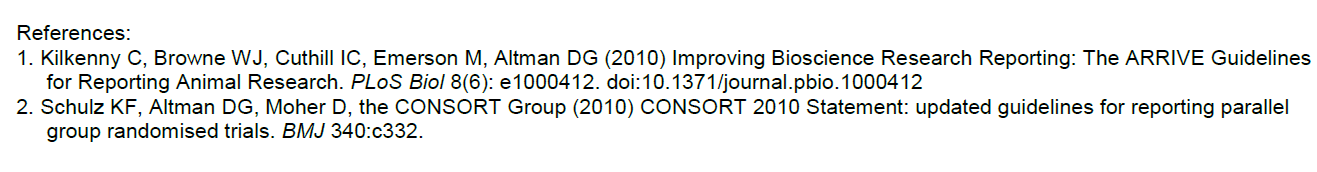

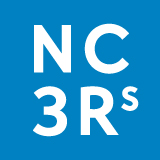

Supplement: S8 Table — Animal Research: Reporting In Vivo Experiments. (DOCX) [file pone.0192643.s008.docx]
